# Accessible independent housing for people with disabilities: A scoping review of promising practices, policies and interventions

**DOI:** 10.1371/journal.pone.0291228

**Published:** 2024-01-25

**Authors:** Sally Lindsay, Kristina Fuentes, Sharmigaa Ragunathan, Yiyan Li, Timothy Ross

**Affiliations:** 1 Bloorview Research Institute, Holland Bloorview Kids Rehabilitation Hospital, East York, Canada; 2 Department of Occupational Science & Occupational Therapy, University of Toronto, Toronto, Canada; 3 Rehabilitation Sciences Institute, University of Toronto, Toronto, Canada; 4 Department of Geography & Planning, University of Toronto, Toronto, Canada; Florida Atlantic University Charles E Schmidt College of Medicine, UNITED STATES

## Abstract

**Background:**

Accessible housing is imperative to enabling independent living for many people with disabilities; yet, research consistently shows how people with disabilities often lack appropriate accessible housing and are more likely to experience unaffordable, insecure, and/or poor quality housing. Therefore, the aim of this study was to understand promising practices, policies and interventions regarding accessible independent housing for people with disabilities.

**Methods:**

We conducted a scoping review that involved searching seven international literature databases that identified 4831 studies, 60 of which met our inclusion criteria.

**Results:**

The reviewed studies involved 18 countries over a 20-year period. Our review highlighted the following key trends: (1) removing barriers to obtaining accessible housing (e.g., advocacy, builders enhancing housing supply, subsidies and financial incentives); (2) policies influencing accessible housing; (3) interventions to enhance accessible housing (i.e., home modifications, smart homes, mobile applications and other experimental devices); and (4) the impact of accessible independent housing on health and wellbeing.

**Conclusions:**

Our findings emphasize the importance of accessible housing for people with disabilities and the urgent need to advance accessible housing options.

## Introduction

People with disabilities, who represent one of the world’s largest minoritized groups, often struggle to find and acquire accessible and affordable housing [1,2]. The United Nations Flagship Report on Disability and Sustainable Development Goals highlights that the housing situation of people with disabilities is a key area of challenge over the next decade [3]. Approximately 22% of Canadians (some 6.2 million people) have a disability and this number is expected to increase rapidly with Canada’s aging population, heightening the demand for accessible housing [4,5]. Thus, the magnitude of accessible housing challenges is likely to escalate unless large scale efforts are made to improve the situation and to address issues in the physical housing environment [6]. In stating accessible housing throughout this paper, we are referring to housing that has been designed or modified to satisfy applicable accessibility requirements and to account for the needs and preferences of people with disabilities.

Although accessible housing policies exist, they often fail to cover the majority of the housing supply [1]. For example, the Center for International Economics [7] estimates that less than 10% of new housing stock has been built to accessible standards. Consequently, many people with disabilities live in housing that does not meet their needs [8] and those who do could face barriers in building relationships with those in their surrounding community (e.g., they may be unable to visit the inaccessible homes of neighbors, friends and family). In Canada for example, people with disabilities are more likely to live in conditions of “core housing need” than the rest of the population, indicating that their housing does not meet a minimum standard of adequacy, suitability, or affordability [9]. Many people with disabilities have specific accessible housing requirements, and at the same time have severely limited housing options [10]. The lack of accessible housing options can be exacerbated for some people with disabilities who need or prefer to live in close proximity to accessible public transit stations, employment opportunities and everyday services (e.g., health care services, grocery stores etc.). Such a lack of inclusive, affordable and accessible housing perpetuates the marginalization and social exclusion of people with disabilities [11].

### Benefits of accessible housing

Accessible housing is critical to the wellbeing of people with disabilities because it can provide them with many health and social benefits for people with disabilities. Indeed, home adaptations are a central part of the rehabilitation process for people with disabilities [12]. For example, research consistently highlights that housing accessibility and affordability are important social determinants of health that are associated with improved health outcomes and quality of life [13–16]. Some research demonstrates that interventions to enhance the accessibility of homes (e.g., home modifications, creating space to accommodate mobility devices and implementing accessible designs) can have positive health and social impacts, such as reduced depression, mortality rates, and falls or injuries, in addition to enhanced social participation [2,17]. Further, having accessibility related home modifications can improve independence, safety, privacy and self-confidence for people with disabilities [8]. Building or adapting homes to an accessible standard could help to reduce costs for health services by decreasing household accidents, the need for institutional care and reliance on other government resources [18]. It is therefore critical to understand the most promising practices, policies and interventions that can help to enhance accessible independent housing for people with disabilities.

### Challenges acquiring accessible housing

Although appropriate accessible housing enables independent living, research shows that people with disabilities often lack suitable housing, are more likely to experience an increased likelihood of living in unaffordable, insecure and/or poor quality housing and are at a higher risk of experiencing homelessness [10,19–21]. People with disabilities often encounter barriers and stigma/discrimination in finding appropriate housing, including learning about available accessible units, securing appointments and receiving reasonable modification requests [22]. For example, policies and practices for renting accessible social housing are often complex and difficult for applicants with disabilities to navigate [23]. Additionally, home builders often lack an understanding of the basic needs of people with disabilities and are reluctant to innovate or increase costs by customizing accessible homes [24,25]. Indeed, there is often insufficient attention paid to design features (e.g., mobility-related, sensory, lighting, sounds, tactile features) that can make housing accessible and liveable for a broad range of needs [1]. The scarcity of accessible housing stems from and is supported by widespread and normalized ableism (i.e., disability-related discrimination), which allows the housing needs of people with disabilities to be ignored. The shortage of accessible housing is disconcerting because the United Nations Convention on the Rights of Persons with Disabilities (Articles 9, 28) states that access to adequate, safe, secure, accessible and affordable housing is a fundamental human right [26]. Specifically, people with disabilities have the right “to choose their place of residence and where and with whom they live on an equal basis with others” [26].

The severe lack of appropriate accessible housing is worrying because it can lead to several poor health and social outcomes [27]. For example, home environments without basic accessibility components can increase the risk of falls, injuries, mortality rates and the use of social services while also restricting social participation, including employment [2,24,28]. Indeed, the physical and social characteristics of housing can contribute to disabling and discriminatory environments for people with disabilities [19,25,29,30]. For these reasons, there is an urgent demand to improve practices to help match the available accessible housing with people with disabilities who need it the most [23]. Synthesizing the promising practices, policies and interventions for increasing accessible independent housing could help to enhance the social inclusion of people with disabilities, while also supporting changes in the dynamics of privilege and marginalization.

### Novelty of this review

This scoping review is timely and significant because many nations, including Canada, where the authors are located, are experiencing a housing crisis, which is expected to worsen with rising rental and development costs, increased interest rates and an ongoing shortage of accessible and affordable housing [8]. People with disabilities are particularly vulnerable to missing out on acquiring affordable and accessible housing and deserve solutions that enhance their access to appropriate housing. Although the literature on the housing needs of people with disabilities is growing, most of the focus is on elderly populations, those with mental health conditions (and risk of homelessness and ‘housing first’ programs), and people with developmental or intellectual disabilities living in group homes or supported housing (e.g., onsite-support and services offered to occupants to maintain their well-being) [2,31–37]. While this research is valuable, there is a critical need to synthesize the most promising practices [38–40], policies [41,42] and interventions (e.g., home modifications [17,27,43–46], universal design [11,47], architectural planning and design [48], smart home technology [49–51], grants and funding [52–54] to enhance accessible *independent* housing for people with various types of disabilities. By doing so, this could help to enhance the social inclusion of people with disabilities, while aiming to shift the dynamics of privilege and marginalization. There remains a concerning lack of knowledge synthesis about accessible independent housing solutions for people with disabilities [8]. Focusing on independent housing is critical because most people with disabilities would prefer to live independently [55]. The findings of this review could help to inform guidelines and recommendations for the most promising practices, policies and interventions to enhance accessible independent housing options for people with disabilities.

## Materials and methods

The research question guiding this review was, what are the most promising policies, practices and interventions for enhancing accessible independent housing for people with disabilities? Our scoping review followed the guidelines outlined by Arksey and O’Malley [56] and subsequently enhanced by Levac et al [57]. The strength of a scoping review is its ability to capture a diverse body of evidence, giving a sense of meaning and significance [58,59]. This type of review can provide a rigorous and transparent method for mapping the size and scope of a research topic while also synthesizing the findings and identifying directions for future research in a short time span [56,59]. Scoping reviews are also useful for assessing the types of evidence that address and inform practice in the field and the way research has been conducted [59]. The protocol of this study was not registered as it is not required for scoping reviews.

### Search strategy and data sources

The search strategy and database selection were developed through consultation with an experienced research librarian, people with lived experience with a disability and knowledge user advisory group (i.e., people with disabilities, social inclusion and housing expert). A series of international searches for peer-reviewed published literature were conducted using the following six databases including: Avery Index to Architectural periodicals, GEOBASE, Engineering Village, PAIS Index, Scopus, and Web of Science. These databases were selected because they are the most relevant to our research question. Our search involved the following key populations, concepts and contexts: *disability* (i.e., disability, disabled persons, functional limitation, physical impairment, mobility impairment, sensory impairment, motor impairment, vision impairment, hearing impairment, wheelchair user), *accessibility* (i.e., accessibility, universal design, built environment, architectural accessibility, facility design construction, adaptation, modification, environment design, smart home technology, adjustment, inclusive design, barrier-free design), and *housing* (i.e., house, housing, home, living environment, residence characteristics, independent living, life span housing) (see S1 File for full search strategy). Minor modifications to the search strategy were made as required within individual databases.

### Article selection

The following inclusion criteria were applied to screen articles for inclusion in this review: (1) a sample of people with disabilities (based on the definition from the International Classification of Functioning, Disability and Health: “an umbrella term for impairments, activity limitations and participation restrictions”) [60]; (2) empirical research (i.e., quantitative or qualitative) that has at least one finding focusing on practices, policies, interventions or solutions to enhance accessible (i.e., a home environment that allows a person with functional limitations to get into and out of, circulate within the home and function independently (2)) and independent housing for people with disabilities; and (3) published from January 2002 to December 2022 in a peer-reviewed journal without language restrictions. Two articles included in our review were translated into English using *Google* translate. The translations were verified by a team member who is fluent in the language. A third team member also read and checked the article to ensure that we derived the appropriate meaning. Additionally, focusing on a 20-year time period is common practice for scoping reviews.

Exclusion criteria included: (1) grey literature (opinions, editorials, books, book reviews and conferences, thesis and dissertations), (2) non-empirical, non-peer reviewed (i.e., grey literature), and (3) research focusing on identifying needs, supported housing, group homes, nursing homes and risk of homelessness among people with disabilities because reviews already exist on these topics [31,32,34–36]. We also excluded (4) non-peer reviewed and grey literature because it is more susceptible to bias [61]. Additionally, empirical peer-reviewed literature is essential for evidence informed decision-making, policy development and identification of gaps in evidence [62,63].

**Fig 1:**
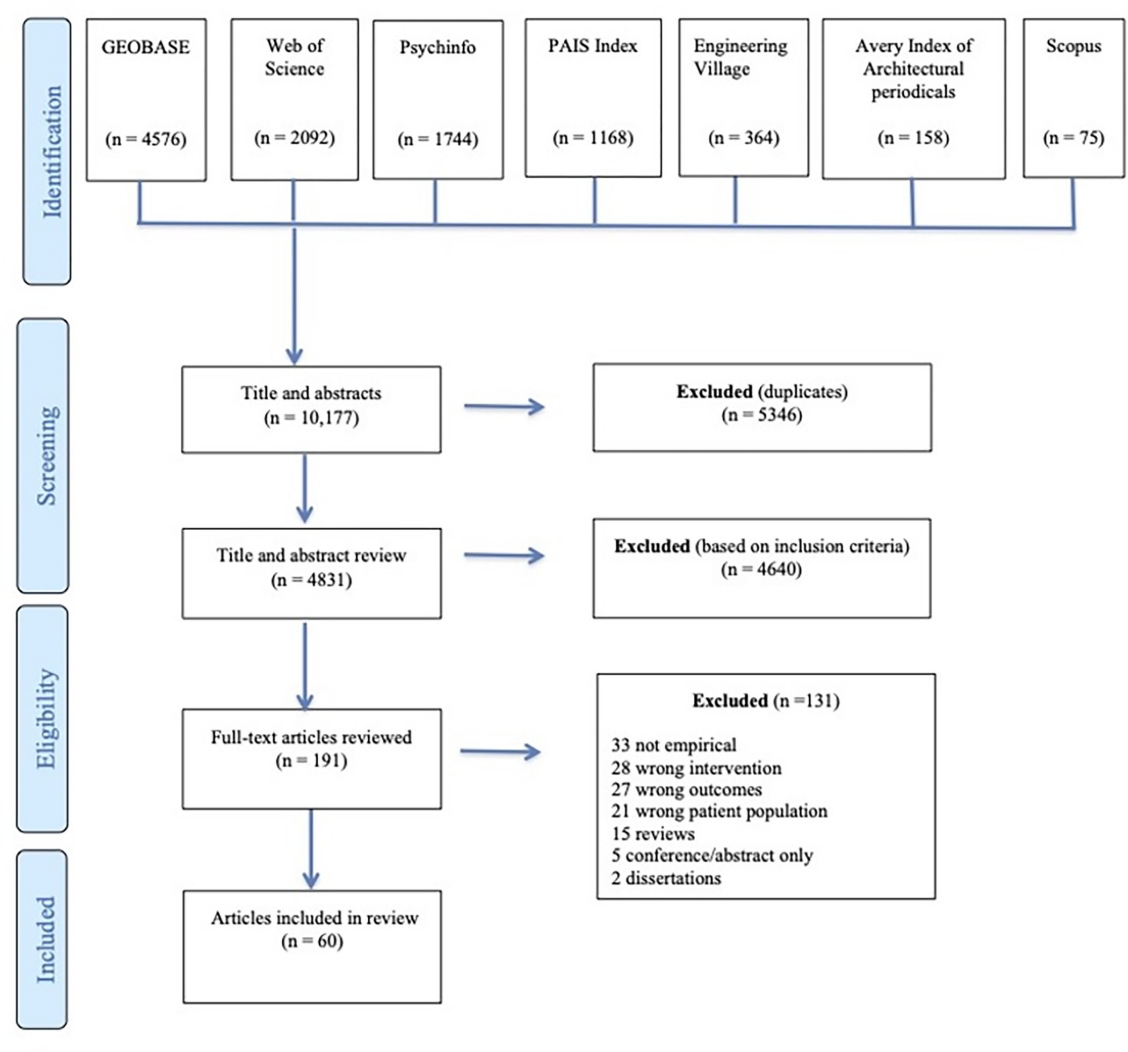
PRISMA flow chart describing the search process and study selection.

After conducting the searches across databases and removing the duplicates, the first author imported the article records into *Covidence*, which is a primary screening and data extraction tool that helps to make the screening process more efficient and transparent. The first author, with expertise in the topic and in review methodology, independently screened all 4831 titles and abstracts, while another researcher screened and verified the excluded studies [59]. Next, two authors screened all of the full-texts (n = 191) that met the inclusion criteria. In the end, 60 articles were identified for our review (see Fig 1). Any discrepancies occurring during the screening process were discussed amongst the research team until consensus was reached. A journal of inclusion/exclusion decisions were kept as part of an audit trail (see also Fi 1 for a list of reasons for exclusion). Journal entries were used to formulate discussion points amongst the research team. We documented the screening process using the Preferred Reporting Items for Systematic Reviews and Meta-Analyses extension for scoping reviews checklist (PRISMA-ScR) (see S2 File) [64].

### Data abstraction and synthesis

Data from all included studies (both qualitative and quantitative) were extracted and compiled by the first author and independently verified by two pairs of authors using a structured pre-defined abstraction form [59]. Data included information about each study (authorship, year of publication, country, recruitment setting, and design), participants (sample size, age, disability type, and social demographics) and outcomes (e.g., practices, policies and interventions for accessible housing). We used a descriptive analytical method to extract relevant information from included articles. Following the Arksey and O’Malley [56] framework, pairs of authors independently charted the findings of each study by type of practice (e.g., practices, policies, interventions) while focusing on the most promising approaches to improve accessible independent housing. The second stage of analysis involved a ‘within study analysis’, which consists of a narrative description of each study’s findings. Then, we compared and contrasted the findings within and between each outcome. Next, we organized the results by the content of the findings within and across the included studies. This was done by grouping the results by topics and themes related to policies, practices and interventions for enhancing accessible independent housing. Then, the first author synthesized the findings across all of the included studies while also highlighting key trends by participant and disability type. We then summarized and analysed the findings within each study category (e.g., quantitative descriptive frequencies and qualitative content analysis. The research team discussed the patterns across the studies until consensus was reached regarding the final themes for the review.

## Results

### Study participants characteristics

Sixty studies met our inclusion criteria. These studies were conducted over a 20-year period, across 18 countries (i.e., Australia, Canada, China, France, Korea, Ireland, Italy, Japan, New Zealand, Norway, Pakistan, Slovenia, Spain, Sweden, Taiwan, Thailand, UK, US) (see Table 1). Some studies focused on specific types of disabilities such as mobility impairments [8,15,65], functional limitations [66], motor disabilities [67], spinal cord injury [49,68], physical disabilities [43,69], vision impairments [70,71], multiple sclerosis [72], while the remainder (and majority) of the studies included other various (unspecified) types of disabilities. Participants included people with disabilities, their families/caregivers, health care providers, architects, home builders, city planners, government decision makers and other key stakeholders involved in providing accessible housing. Among the (33/60) studies describing the gender composition of their samples, 14 involved women majority samples [8,46,49,50,65,69,72–79], nine had men majority samples [43,46,67,68,80–84] and 10 had approximately equal gender representation [51,66,71,78,79,85–89]. Among the few studies (7/60) describing the racial or ethnic composition of their samples, six included a majority of white participants [50,72,77,81,86,90] and one had a mixed ethnic representation [82]. Ages of the samples ranged considerably with 17 focusing on elderly/older adults [46,53,66,68–70,73,74,76,78,80,81,83,87,91–93], 12 examined adults [8,43,49, 65,71,72,75,79,82,86,88,94], six included a wide age range [11,51,77,85,89,95], while three focused specifically on children/youth and their caregivers [67,84,96]. Of the studies (26/60) reporting on housing location, 11 focused on accessible housing in urban / suburban areas [53,69,74,75,77,86,87,95,97–99], one in a rural area [100] and 14 in mixed locations [8,23,43,67,68,73,76,81,82,88,90,91,96,101]. Less than half of the studies (25/60) described housing types, which included single-family homes, apartments and multi-family housing. Fourteen studies involved mixed representation of housing status [43,46,68,72,73,75,77,83,85,89,95,96,100,101]. In six studies most participants owned their own home [8,69,72,76,84,87] and in three studies the majority rented [23,65,66]. In two studies most participants lived in subsidized housing [70,86].

**Table 1:** Overview of accessible practices, policies and interventions.

	Sample Characteristics	Objective	Design and Analysis (Theory)	Key Findings*
				*Practices*
Alonso-Lopez 2020 (Spain) [108]	23,176 participants (mean age 64.9 years) •Gender, age, disability type, and location and type not reported	To explain how some home expenditure decisions are in practice adaptive behaviors that complement public programs	Secondary analysis of Spanish survey of disabilities (theory: ecological theory of aging)	•Some socio-demographic conditions (age, sex) do not determine the realization of home adaptations •Households from bigger cities, richer regions, with more and severe disabilities are more likely to spend on adaptations •Environmental and functional factors are most relevant in determining adaptations
Anderson et al. 2020 (UK) [23]	28 households with a disabled person (mix of urban and rural locations) •Housing tenure: 57% socially rented; 17.8% privately rented; 14.2% home ownership; 7% tied accommodation; 3.5% staying with family •Gender, age, disability type, not reported	To examine access to social rented housing as a route to independent living through lettings practice for accessible and adapted homes	Interviews and observations (theory: social model of disability)	•Effective matching of disabled applicants to accessible properties involved reletting vacant properties, recovery of properties no longer occupied by a disabled person, nominations from registered social landlords and letting newly built units •Having a single contact to help them through the process; along with up-to-date property information an option for virtual property viewings
Aplin et al. 2013 (Australia) [85]	55 participants (36 clients with various disabilities; 5 parents of children receiving services, 13 spouses of clients, 1 carer); 55% women; aged 25–87 •Housing tenure: 38.1% home ownership; 38.1% government housing •Location not reported	To understand what aspects of the home environment impact home modification decision-making	Interviews (theory: not used)	•4 dimensions of the home commonly affected home modification decision making including personal (safety, privacy sense of freedom, independence), societal (costs, service provider restrictions and government standards for public access), physical (space within the home) and temporal (health status, future growth of family) dimensions of home •Additional dimensions that affect home modification and decision-making and social (visitability) and occupational (self-care, domestic activities) dimensions
Aplin et al. 2018 (Australia) [84]	4 families of children with disabilities living in their own home (mean age 49.2 years for caregivers; 10.5 years—children; 25% girls) •Housing tenure: home ownership •Location not reported	To investigate the experience of home for parents and carers of children with disabilities	Interviews (theory: not used)	•Home modifications and aspects of the home environment helped to make everyday life easier. •Location of the home, appropriate home modifications and planning shaped the experience of home for parents of children with disabilities
Danko 2007 (US) [100]	110 people with disabilities or family members or those working with them •Housing tenure: owned and rental in a rural state •Gender, age, disability type not reported	To identify opportunities and barriers for people with disabilities in obtaining accessible, affordable housing in West Virginia	Surveys and focus group (theory: not used)	•Location is a major barrier to finding accessible housing •Affordable and accessible housing is often in undesirable neighborhoods •Collaborative partnerships with builders, money lenders, landlord associations to share information about universal design and accessibility •training on how to interact with disabled people regarding design techniques •Mountaineer mortgage plus renovation program
Denizou (Norway) 2016 [113]	3 architects •Demographics, housing tenure and location not provided	To identify approaches that strengthen universal design beyond accessibility requirements	Qualitative case study and interviews (theory: not used)	•Architects consider accessibility a natural part of their skills •Knowledge of regulations and guidelines helps creativity •Many struggle to interpret the building code and inconsistent rules
Fange and Iwarsson 2007 (Sweden) [78]	131 people with various disabilities living in ordinary housing with housing adaptations; median age 75; 67.1% women •Housing tenure and location not reported	To understand and problematize challenges in the process of developing strategies for housing adaptation evaluations feasible for municipality contexts	Observation home visits using the Housing Enabler, Usability in my home, instruments (theory: person environment interaction)	•Some challenges with accessibility assessments
Goodwin et al. 2022 (Australia) [8]	145 (112 with mobility impairment; 33 family members) •72.5% of sample under 65 years; 62.1% women •78% lived in private home owned themselves or rented from a private landlord or public authority •27.6% lived in accessible housing •62% lived in metropolitan region	To explore the perspectives of people with mobility impairments and accessible housing modifications	Survey (theory: not used)	•Common modifications included step-free entrance, wider internal doors and corridors, level access •Having an accessible home promoted social inclusion, increased functioning and independence and quality of life
Haak et al. 2015 (Sweden) [66]	26 people with functional limitations (54% men); majority 65+ years •42% lived alone •77% lived in apartment; 23% single family home •rural/urban location not reported •15 professionals; 20 experts (demographics not reported)	To understand the needs and expectations of housing options for people aging with disabilities	Participatory design (theory: ecological model of aging)	•Information barriers on accessible housing, housing adaptation benefits, and cost
Helle and Brandt 2009 (Sweden) [112]	106 cases (accessing accessible housing) •Demographics, housing location and tenure not reported	To develop a content valid cross-Nordic version of the housing enabler and investigation of inter-rater reliability	Housing accessibility assessments (theory: not reported)	•Agreement on personal and environment barriers of the instrument exceeded 80%
Hoffman et al. 2017 (US) [86]	116 housing voucher users (subsidized rental housing in urban areas) •mean age 50.8; 52.3% women; 36.7% non-white (disability type not provided)	To evaluate the effect of housing vouchers on transition rates among nursing home residents eligible for non-elderly disabled housing choice voucher program	Secondary analysis of administrative data (theory: not used)	•Housing voucher program increased community transition rates by 8.7% •Housing subsidies were effective in helping nonelderly people with disabilities living in nursing homes to make a transition to independent living
Kaminski et al. 2006 (US) [97]	•sub-urban area •Demographics and housing type not reported	To explore visitability in Irvine, California	Case study (theory: not reported)	•The voluntary approach of visitability of homebuyers was not working as expected •Builders began to voluntarily include visitability components
Lehning 2011 (US) [98]	62 city planners •urban housing •housing tenure and other demographics not reported	To examine characteristics associated with city government adoption of community design, housing and transportation innovations that could benefit older adults	Surveys and interviews (theory: not used)	•24.2% accessory dwelling unit; 30.6% developer incentives for housing units; 24.2% incentives to make housing accessible; 21% home modification assistance •Advocacy is an effective strategy to encourage city government adoption of innovations •Younger individuals with disabilities are more active in advocacy •Cities that experienced public advocacy had a higher percent of the population with a disability had an increased odds of adopting community design innovations •Existence of a policy entrepreneur was positively associated with innovation adoption
Lien et al. 2016 (US) [90]	50 home assessments (rural and urban locations; various housing types—single family, multifamily housing) •85.2% white; 61.6% owner-occupied •mean age and gender not reported	To adapt the environmental component of the Swedish housing enabler for valid use in the US	Assessing content validity of Swedish housing enabler instrument for use in US (theory: person- environment fit)	•Swedish housing enabler instrument was valid and reliable for use in the US
Luther et al. 2020 (Sweden) [73]	241 housing adaptation clients (mean age 75.1 years; 63.1% women); •various types and severities of disabilities •64.3% larger urban area, 21.6% smaller urban area, 13.7% rural •housing tenure: 54.4% apartment, 40.2% single family house, 1.7% other	To identify and validate housing adaptations client profiles	Survey and interviews (theory: heterogeneity of characteristics)	•People applying for housing adaptations are a heterogenous group with different needs •5 client profiles: 1) older adults with low level of disability; 2) older adults with medium level of disability; 3) adults with low level of disability; 4) adults with high level of disability; 5) older adults with medium level of disability and moderate cognitive impairment
Morgan et al. 2016 (UK) [96]	35 families with a disabled child •Various locations and housing tenures •Gender, mean age, disability type not reported	To investigate family and professional perspectives of home adaptations for disabled children	Interviews and survey (theory: not used)	•Having input into the adaptation process was beneficial for a satisfactory home adaptation •Themes included: constraints imposed by non-adapted homes; choices and lack of choice related to home •Meaning of home and family life; home as social space; home also represented social support for other family members
Mukaino et al. 2020 (Japan) [80]	60 people with a disability (37% neurological disease, 13% orthopedic diseases, 15% other diseases; 60% men; mean age 70.2) •Housing tenure and location not reported	To identify any mismatch between an individual’s abilities and their home environment	Evaluation of home environment checklist (theory: not reported)	•The home environment checklist had an excellent inter-rater reliability and validity
Nord et al. 2009 (UK) [69]	19 (6 clients with physical disabilities; 66% women; mean age 67.8; 83.4% owner (80% house; 20% flat); 16.6% tenant in sheltered housing); 4 occupational therapists, 6 grant surveyors and 3 builders •Urban location	To explore what constitutes good communication and how communication occurred in the design of home adaptations	Interviews (theory: framework of community practice)	•Professionals constituted a community of practice with good communication •Client involvement in the design (bathroom adaptations) was limited and the plan drawings were not effective tools in communicating with them •Clients were interested in the aesthetics aspects of adaptations
Norin et al. 2021 (Sweden) [68]	122 older adults with spinal cord injuries (29% women, mean age 63 years) •77% live in dwellings with housing adaptations •45% lived in a flat; 55% single-family house •63% urban; 37% rural	To investigate housing adaptations and current accessibility problems in older adults with spinal cord injuries	Home visits (Housing enabler instrument) (theory: person, environment fit)	•Kitchens, entrances and hygiene area were common locations for housing adaptations and environmental barriers that generated accessibility problems •Most common adaptations were ramps, wheelchair-accessible stovetops, ceiling lifts •Wall mounted cupboards and high shelves, inaccessible storage areas, and lack of grab bars generated most accessibility problems
Pettersson et al. 2008 (Sweden) [46]	73 people with disabilities approved for modifications (mean age 75.7; 66% women; 89.7% apartment; 10.3% single family home) •Comparison group: 41 people with disabilities waiting for approval •mean age 74.6 years; 70% women; 81% apartment; 19% single family home)	To examine the impact of home modifications on self-rated ability of everyday life from people aging with disabilities	Database of home modifications (theory: not used)	•Those who received home modifications had a significant improvement in their self-rated ability in everyday life compared to comparison group •Those who had home modifications had less difficulty and increased safety, especially self-care tasks in the bathroom and transfers such as getting in and out of the home
Pettersson et al. 2012 (Sweden) [79]	4 people with disabilities (neurological disorder, functional limitations) needing housing adaptations (1 man, 3 women) •mean age 55.5; housing tenure and location not reported	To explore clients’ experiences of housing adaptations over time in relation to housing and health	Interviews / case study (theory: person environment activity)	•Housing adaptations helped participants to live independently •Design or physical expression of housing adaptions (i.e., both functional and aesthetic aspects) was important •The way of contacting professionals (clinicians, municipality, workmen) affected their experience of housing adaptations as a process •Interprofessional team to develop the best housing adaptation solutions
Pettersson et al. 2018 (Sweden) [103]	609 dwellings (416 apartments in multi-dwelling blocks and 193 single-family houses in urban areas); aged 45–93 years; various disabilities •mean age, gender not reported	To estimate the effects of targeted elimination of environmental barriers in ordinary housing stock in Sweden and the effects on accessibility at a population level	Secondary analysis of Swedish housing data (theory: person environment fit)	•Estimated targeted elimination of barriers could have the largest accessibility effects for the more complex functional profiles •Effects are larger for one-family houses and for all types of dwellings built before 1960
Rooney et al. 2018 (UK) [70]	12 visually impaired (6 occupants of sheltered housing; mean age 82.6) 6 from lifetime homes, mean age 58.5 •Gender and location not reported	To understand the experiences of visually impaired older people living independently at home	Interviews (theory: social emersion; and salutogenic theory)	•People with vision impairments use adaptative strategies to modify their home •Themes included negotiating priorities (enhancing homes; housing priorities; compromise; home sweet home); understanding visual impairment (adverse reaction, approach to visual impairment, positive frame of mind, relationship with others, needs of visually impaired people) •Those who coped well with moving used various resources
Roy et al. 2008 (Canada) [67]	11 parents (8 women, 3 men) of children with motor disabilities (6 boys, 4 girls, mean age 13; 54.4% urban; 36.4% rural) •Housing tenure not reported	To explore the experiences of parents of children with motor disabilities through the home adaptation process	Interviews and focus groups (theory: model of competence)	•Had a beneficial impact for child’s independence and self-esteem •Respect for temporal aspects, safety and parent’s roles •A need for information and appearance of the home •Many parents had to make their own adaptations
Sukkay 2016 (Thailand) [106]	9 participants with mobility disabilities •Demographics, housing location and tenure not reported	To identify the body of functioning of a group of people with mobility disabilities; to perform post-occupancy evaluation of this group and their houses	Survey, observation and interview (theory: not used)	•Room dimensions and furniture materials had an impact on accessibility •Bathrooms and bedrooms were the most difficult to access and to perform daily activities
Wellecke et al. 2022 (Australia) [91]	144 occupational therapists (76.3% conducted home assessments for older people in their own home; 9% for younger people with disabilities; 6.9% for outpatients; representation across urban and remote regions •Age, gender and housing type not reported	To investigate accessible housing design features and home modifications	Survey (theory: not used)	•Most important housing design features include step-free access to the dwelling, large step-free showers and bedroom space on ground floor •Importance of reinforcing bathroom walls and grab rails
Whitehead and Golding-Day 2019 (UK) [87]	21 older adults (mean age 74.9; 57% women; owned council housing; urban area) •10 included in expedited adaptations group; 11 in wait list controls •5 carers (mean age 72 years)	To explore the lived experience of bathing adaptations in the homes of older adults and their carers	Interviews (theory: person-environment fit)	Themes related to the benefits of bathing adaptations: ease of use, feeling safe, clean, independent, choice and control, confidence and quality of life
				*Policies*
Callaway et al. 2022 (Australia) [54]	8 representatives from government injury and disability insurers •Demographics, location and housing tenure not reported	To identify the issues and trends; factors for decision-making and service impacts related to housing and support for people with disabilities and high daily support needs	Focus group (theory: not used)	Themes included: influences on decisions to fund housing and/or supports, identify good housing solutions, evaluate cost and benefit of housing and support investments (e.g., modifications); developing future investment in housing and support
Chu and Shen 2022 (China) [105]	30 provinces in China •Demographics, housing tenure and location not reported	To explore factors that facilitate or impede the adoption of policy innovation on major housing adaptation by Chinese provincial governments	Policy case study and event history analysis (policy innovation and diffusion theory)	•Diffusion mechanisms can significantly facilitate or impede the adoption of major housing assessment policy innovations by governments •Policy adoption by neighboring governments helps facilitate policy adoption by nonadopters
Dunn 2002 (Canada) [104]	200 key informants (government officials responsible for disability policy) •Demographics, housing location and tenure not reported	To describe the evolution of government independent living policies and programs for Canadians with disabilities	Survey and secondary analysis of statistics Canada data (theory: not used)	•Barrier free housing can help people to live independently •8/12 provinces and territories had accessible building codes by the end of the decade, which included barrier-free standards of apartment buildings •7/10 provinces and territories developed their own housing adaptation programmes and 10 had home improvement programmes that often included funding for home modifications
Fallon and Price 2020 (US) [81]	100 low income housing-tax funded properties; 51% urban; 49% rural (80% men; mean age 64; 59% disabled; 67% white, 27% Black, 5% other race •Housing type not reported	To examine the preferences, satisfaction and perceived ability to age in place for Ohioans living in housing subsidized by the low-income housing tax credit program	Survey (theory: not used)	•Residents prioritize affordability and safety •Other preferences vary by age and mobility disability •residents showed high levels of satisfaction with housing and neighborhood components; however seniors demonstrated higher overall housing satisfaction and perceived ability to age in place •Older adults with a mobility disability preferred a home that is accessible for people with disabilities •Challenges for affordability and accessibility for residents in developments subsidized by the housing tax credit program
Heller et al. 2022 (Sweden) [88]	11 housing stakeholders (6 men, 5 women; median age 43 years) •Mix of rural and urban •Housing type not reported	To gain an in-depth understanding of how municipalities addressing housing accessibility issues and explore what types of policy solutions they consider for the future	Interviews (theory: not used)	•Housing accessibility involves the following themes: •Organizational policies (dialogue and collaboration with different actors); •Economic policies (financial incentives for different housing provision actors, financial incentives for individuals to stimulate relocation, subsidies targeting improvement of current housing stock, and interventions focusing on social division) •Research and development policies (measures focusing on technology, development of new design solutions) •Preventive policies (accessibility inventories as a basis for future measures; preventive measures that ensure accessibility is addressed before need arises) •Housing construction and design policies (individual housing adaptations, refurbishment, renovation and maintenance) •Legal policies (sharpen housing and building legislation)
Imrie 2003 (UK) [102]	•210 housebuilders •20 national housebuilders •Demographics not provided	To describe and evaluate reactions of house builders in England and Wales to government regulation concerning disabled people’s physical access to new housing	Survey and interviews (theory: not used)	•Builders believe that the market for accessible housing is small and insignificant •41.9% of house builders already provide a sufficient supply of accessible dwellings
Semeah et al. 2019 (US) [82]	39 Veterans with disabilities (mental health, musculoskeletal, trauma, neurological, other) •82% male; aged 18+; 41% white, 23% black, 36% other race •61% urban; 34% rural •Housing type not reported	To identify facilitators and barriers to finding an maintaining rental housing	Survey (theory: biopsychoecological model)	•Housing policy (Fair Housing Amendments Act; subsidized housing, and reasonable modifications under the fair housing amendments act, service animal guidelines) was a facilitator and barrier to accessing rental housing
				*Interventions / solutions*
Aplin et al. 2015 (Australia) [89]	55 participants (36 clients with various disabilities; 5 parents of children receiving services, 13 spouses of clients, 1 carer); 55% women; aged 25–87 years •50% own home, 50% government housing •Location not reported	To explore the impact of home modifications on clients and their family’s experience of home	In-depth interviews (theory: not used)	•Most participants reported positive changes to their experience of home after modification which was influenced by 5 dimensions of home environment (personal, occupational, physical, temporal and social) •Positive impact of modifications included enhanced sense of safety, relief and reduced levels of anxiety while negative impacts resulted in unsafe situations-Outcomes of modifications were influenced by workmanship, consultation or involvement in decision-making and societal dimension of the home environment
Bishop et al. 2015 (US) [72]	5082 adults with multiple sclerosis (mean age 54.1; 77.8% women; 96% white, 1.9% Black, 1.3% Hispanic, 1.5% other race) •19.5% urban; 54.1% suburban 26.4% rural •77% owned their house, 15.5% rent a house or apartment, 4.1% live with a friend or family	To evaluate the prevalence of housing modification and devices among Americans with multiple sclerosis	Survey (theory: not used)	•Small number of changes comprised the majority of modifications people used or needed •most prevalent modifications involved the bathroom •Other modifications included entryways, living area, interior stairs, doorways, bedroom, kitchen and flooring •Those with greater degrees of mobility limitation had an increased likelihood of making a housing modification
Busby and Harrison 2018 (Ireland) [99]	2^nd^ year architectural students applying universal design, suburban typology of detached houses •Demographics not reported	To engage architectural students in valuable universal design exercises with user-experts	Live project, case study (theory: not used)	•Education exercise shapes students thinking about universal design
Carnemolla and Bridge 2014 (Australia) [11]	89 recipients of home modifications (aged 52–96) •Demographics; housing location and tenure not reported	To explore the potential for home modifications to impact ageing well	Survey (universal design theory)	•Improved health related quality of life is associated with home modifications •home modifications provide safety, confidence, mobility throughout the home, independence, and community participation •Home modifications play a dual role of addressing broader societal concerns about accessible housing and care demand and individual needs and supporting aging needs beyond the physical environment
Chen et al. 2007 (Taiwan) [49]	12 (66% women, aged 26–47 years) -control group: 6 without disabilities, 6 with spinal cord injury •Demographic, housing location and tenure not reported	To propose an eyeglass-type infrared-based home appliance control system for spinal cord injured with tetraplegia	Design system for home appliance control system (headset, infrared transmitting module and infrared receiving/signal processing module; main controller and microprocessor) (theory: not used)	•Home appliance control system provided advantages of convenience, accuracy and sanitation for people with spinal cord injury •No significant differences in the accuracy and average time cost of the control and experimental groups •Appliance system helped them to live more independently
Costa et al. 2021 (Italy) [12]	362 people with disabilities needing home adaptations •Demographics, housing location and tenure not reported	To describe the strategy and impact of a home adaptation project	•Home site surveys and interviews (theory: habitation; person environment relationship)	•Home adaptations affect how disabled people live in their homes; allowed them to have more autonomy, promoting safety, overcoming stigma, re-defined family roles, opportunities for the senses •Increase in accessibility is the most common effect of adaptations in terms of affordances •Adaptations increased safety at home
De-Rosende-Celeiro et al. 2019 (Spain) [74]	193 urban community-dwelling older adults with disabilities (cognitive impairment, osteoarthritis, multiple disabilities (median age 84; 69.7% women) •Housing type not reported	To explore the use of assistive products to promote functional independence in self-care activities in the bathroom; and to explore the role of environmental factors in predicting implementation of bathroom adaptations	•Cross sectional design; home assessments (theory: matching person and technology model)	•The number of categories of assistive products used in the bath was positively associated with the independent performance of the transfer •Social functioning was significantly associated with the implementation of a bathroom adaptation •Four personal factors, including osteoarthritis in lower limb, Parkinson’s disease, rehabilitation intervention and hospital stay, were significantly related to a bathroom adaptation •Social risk was significantly lower in participants who made an adaptation of the bathroom
Ding et al. (US) 2021 [50]	15 practitioners with experience in smart home technology and providing home modifications to people with various types of disabilities (60% women; 93.3% white) •mean age, housing location and type not reported	To examine how mainstream smart home technology is becoming affordable and relevant for improving environmental control and independence of people with disabilities	Survey, and interviews (theory: not used)	•Common practice in smart home service delivery (Importance of client centred approach, informal service delivery process, trial and error in setting up technology) •Factors affecting delivery: (influence of client familiarity with technologies and existing use of technology on service delivery; importance of conducting needs assessment and ability assessment) •Important considerations of smart home service delivery: (demonstrating or trialing the technology; device customization and client training) •There are potential benefits of smart home technology for people with disabilities (improved independence, safety, social connection)
Douglas et al. (Australia) 2022 [75]	15 people with a disability (neurological disorder or cerebral palsy, complex needs), aged 18–65; mean age 44.2; 60% women •26.7% shared supported accommodation, 40% private home, 13.3% residential aged care, 6.7% vulnerable housing •Urban area	To assess the change in individual outcomes for people with disability and complex needs after moving into newly built individualized apartments in the community	Pre and post self-report measures (visual analogue scale, Warwick-Edinburgh mental wellbeing scale, community integration questionnaire) (theory: not used)	•Significant improvements (large effect) in well-being and community integration and a trend towards improved health, reduced support needs (average decrease of 2.4 support hours per day)
Fange and Iwarsson 2005 (Sweden) [76]	131 people with various disabilities living in general housing (67.1% women; median age 75) •56% single family home; 47% apartment •Mix of rural and urban locations	To investigate longitudinal changes in housing accessibility among clients receiving grants for housing adaptations	Pre-post housing assessments (housing enabler and usability in my home instrument) (theory: person environment interaction)	•Accessibility and usability improved significantly •Number of physical environmental barriers decreased and dependence on mobility devices
Fisk and Raynham 2014 (UK) [101]	80 rehabilitation workers and occupational therapists •Mix of rural and urban locations; various types of housing •Demographics not provided	To explore the importance of lighting for people with sight loss	Focus groups (theory: not used)	•Need for lighting should be appropriate, sufficient, even, adjustable, sustainable and energy efficient, simple and adaptable
Granbom et al. 2017 (Sweden) [83]	9 caregivers of people with various disabilities applying for a housing adaptation grant (33% women, mean age 68) •Mixed dwelling types; •Location not reported	To describe the cohabitants’ expectations and experiences of how a housing adaptation could impact their everyday life	Interviews (theory: not used)	•Cohabitants’ expectations and experiences on the housing adaptation were influenced by their perception of their own and their partner’s health and wellbeing •Expectations and experiences involved: partners’ activities and independence; cohabitants’ everyday activities and caregiving; couples’ shared recreational activities and housing decisions
Greiman et al. 2022 (US) [77]	195 people with mobility disabilities (62.6% women, mean age 53.9; 76.4% white, 16.9% Black, 7.7% Native, 3.1% Hispanic) •urban and suburban area •26.6% home ownership, 67.2% rent, 6.3% other •42.2% single family home, 9.4% town home, 41.2% apartment, 7.3% other	To explore the effect of a consumer-driven home modification intervention on community participation for people with mobility disabilities	Randomized control trial where the intervention was the Home Usability Program (brief community engagement questionnaire) (theory: not used)	•Home usability program positively affected the community participation of people with mobility disabilities •39.5% increase in social and recreational activities in the intervention group compared to controls •Changes to the bathroom was most frequently chosen home modification, followed by changes to facilitate cleaning, improve mobility in the home, safety and improvements to home entrance
Helle et al. 2015 (Sweden) [92]	30 people aged 65+ •Gender, mean age, disability, housing type and location not reported	To combine apps targeting professions and senior citizens to improve housing accessibility and influence housing provisions	User-involvement participatory and user-centered design including field notes and recordings; questionnaire diaries and focus group interviews (theory: not reported)	•A user-friendly and acceptable prototype was developed •Design features differed between the groups—seniors wanted simple and easy to use interface; professionals wanted design that supported extended functionalities
Jonsson et al. 2018 (Sweden) [4]	•Demographics, housing type and location not described	To develop and evaluate a new decision support system to inventory and support decision-making for improved accessibility in multi-family housing in Sweden	Co-design of an app prototype (housing enabler) (theory: ecological model of aging)	•App enables professional inventory of environmental barriers and work for accessibility improvements •Co-designed to fit the practice context and target users’ needs and expectations
Kosmyna et al. 2016 (France) [94]	14 (12 healthy participants aged 23–45; 2 with a motor disability aged 25–27) •Gender, mean age, housing type and location not reported	To design a control mechanism for smart homes based on brain computer interface	Experiment (usability and feasibility questionnaire) (theory: not used)	•Users were able to control lighting, tv set, coffee machine and shutters of the smart home •Healthy subjects had 77% task accuracy and disabled had 81%
Kuboshima and McIntosh 2021 (New Zealand) [55]	16 participants who use a walker •Demographics, housing type and location not reported	To investigate the perceptions of spatial use of older adults using a walker	Interviews and observation (theory: not used)	•Design elements and considerations: layout of space that avoids needing to turn around in small, enclosed spaces; remove ground-level floor differences; minimize interior doors; avoid sharp corners in circulation; attention to the location of door handles; enough space in a bathroom and communal area to accommodate a walker; L or I shaped kitchens; garage and parking should allow enough space for walker
Kutintara et al. 2013 (Thailand) [71]	10 people with vision impairments (4 men, 5 women, aged 24–49) •Housing type and location not reported	To design and evaluate a kitchen for people with visual impairments	Interviews and observation (theory: not used)	•Those who attended the cooking courses were able to cook safely in the kitchen •Participants preferred sliding cabinet doors, shelf inside, D shaped drawer pulls •Liked the contrasting colored strip edges of kitchen counter
Lee et al. 2018 (Korea) [43]	80 people with physical disabilities (60% men; mean age 46.4 years; 40 living in a rural area; 40 in an urban area) •37.5% lived in single family house, 62.5% other housing type	To identify the potential effects of home renovation on independence promotion of physically disabled Koreans living at home	Survey (theory: not used)	•Most renovation needs involved safety handles and grab bars, removing door sills and stepped pulleys and replacing wall paper and finishing materials •More requests for forming lighting and thermal environments and improving the system for humidity control in rural areas •Participants were satisfied with their renovation experience (3.42/5.0)
Mattie et al. 2016 (Canada) [65]	30 people with mobility limitations (60% used a wheelchair); mean age 47.9 years, 60% women •60% lived in apartment or condo •Housing location not reported	To evaluate end user perspectives of existing home access solutions (HAS) and a newly designed experimental device (ARISE)	Cross sectional design, observational evaluations (theory: not used)	•5 home access features were considered important by 90% of participants: ease of use, ability to use independently, reliability, safety and security •Over 80% of participants were satisfied with the ramp and the platform lift •ARISE prototype was rated as the most preferred device by most participants
Ocepek et al. 2013 (Slovenia) [51]	59 people with disabilities (amputation, neuromuscular, spinal cord injury, rheumatic disease, multiple disabilities; aged 24–81 (30 men, 29 women) •Housing type and location not reported	To evaluate the treatment of smart home Independent Residing enabled by Intelligent Solutions (IRIS) in terms of functional independence and occupational performance and satisfaction	Quasi-experimental design (theory: not used)	•Statistically significant improvements in functional independence scores and Canadian Occupational Performance measure scores
Oyegoke et al. 2022 (UK) [110]	•Demographics, housing type and location not reported	To develop an innovative smart solution to streamline housing adaptation process to prevent delays for disabled people	Co-development and focus groups (theory: not used)	•Adapt-ABLE approach uses optimization techniques through an information technology system for streamlining the process •Allows for development of a preventive measure that can assess suitability index of homes for the occupants
Rooney et al. 2016 (UK) [107]	13 key stakeholders (clinicians, researchers, housing officers) •Demographics, housing type and location not reported	To explore perceptions on the suitability and effectiveness of lifetime homes standards for those with visual impairment	Interviews (social model of disability)	•Having lifetime homes standards offers benefits to visually impaired residents •Benefits include: future proofing features, extra space, sight loss features •Limitations of lifetime home standards: box ticking, collaboration between stakeholders, knowledge of sight loss, awareness of standards, design restrictions
Tayyaba et al. 2020 (Pakistan) [111]	•Demographics, housing type and location not reported	To assess a fuzzy-based approach using internet of things devices for smart home for blind people	Simulation assessment (theory: not used)	•A model smart home comprising sensors and antennas generates warning signals about obstacles and navigates the user to move around the house safely
Tongsiri et al. 2017 (Thailand) [109]	43 people with disabilities •Demographics, housing type and location not reported	To assess home modifications for people with physical disabilities	Home modification assessments (theory: not used)	•After the home modifications participants reported reduced difficulties in all areas except for those with severe degrees of difficulties •Quality of life improved
Tsuchiya-Ito et al. 2022 (Japan) [53]	10,372 people with various disabilities (62.6% women, aged 65+) •Suburban area; housing type not reported	To examine the utilization of housing adaptation grants in terms of implementation and costs	Secondary data analysis (theory: Andersen’s behavioural model)	•Housing adaptations were implemented among 15.6% of the sample and median cost was $1287 (US) •Those with lower disability levels, lower extremity impairment or poor balance were more likely to implement housing adaptations

*Note that we only report on findings related to our research question.

The methodological designs of the reviewed studies involved qualitative (i.e., interviews, focus groups, observations and assessments, case study, participatory design) and quantitative (i.e., surveys, secondary analysis of existing datasets, experiment, quasi-experiment and co-design) methods. Among the studies (22/60) incorporating a theoretical framework they included person- environment interaction/fit, ecological model of aging, heterogeneity of characteristics, framework of community practice, neoliberal spaces of exception, social emersion, salutogenic theory, model of competence, diffusion theory, biopsychoecological model, universal design theory, habitation, matching person and technology model, social model of disability, and Andersen’s behavioral model.

Our review identified four key themes in the literature: (1) removing barriers to obtaining accessible housing; (2) policies influencing accessible housing; (3) interventions to enhance accessible housing and (4) the impact of accessible independent housing on health and wellbeing.

### Theme 1: Removing barriers to obtaining accessible housing

Eighteen studies described approaches to removing barriers to obtaining accessible housing such as advocacy, builders enhancing housing supply, subsidies and financial incentives, in addition to effective communication and collaborative partnerships.

Two studies explained the importance of advocacy to help reduce barriers in obtaining accessible housing [98,100]. For example, in a survey of city planners in the US, Lehning [98] found that advocacy was an effective strategy to reduce barriers and encourage municipal governments to adopt housing innovations. In particular, their study discovered that cities experiencing public advocacy or having a higher percentage of the population with a disability had increased odds of adopting community design innovations [98]. Another study [100] highlighted that in their effort to increase the supply of accessible homes, people with disabilities in the US needed to advocate and appeal to the capitalist side of home builders in advocating for universal design by emphasizing its profitability [100].

#### Builders enhancing the supply of accessible housing

Although many studies mentioned increasing the supply of accessible housing, three studies focused on this directly [97,102,103]. For example, two studies described how home builders facilitated the supply of accessible housing [97,102] and one study focused on the removal of barriers in ordinary housing stock [95]. For instance, Kaminski et al. [97] explored the visitability of houses in one area of California and explained that builders began to voluntarily include visitability components in their houses, which could have resulted from a marketing campaign targeted toward builders to make accessibility features more attractive. Additionally, Imrie [102] evaluated the reactions of home builders to government regulation concerning disabled people’s access to new housing in England and Wales where 41.9% of home builders reported they provide a sufficient supply of accessible dwellings [102]. Meanwhile, Pettersson et al. [95] estimated the effects of targeted elimination of environmental barriers in ordinary housing stock in Sweden and reported the largest effects were for multi-dwelling blocks and single-family houses (especially for houses built before 1960).

#### Subsidies and financial incentives

Seven studies described the use of financial incentives or subsidies to increase the supply of availability of accessible housing [53,76,81,86,88,98,100]. For example, Lehning [98] explained how advocating for accessible housing innovations amongst city planners in the US included financial incentives (i.e., incentives to make housing accessible, developer incentives for housing units). Meanwhile, Hoffman et al. [86] evaluated the effect of a housing voucher program for non-elderly people with disabilities in the US. They reported that the program provided housing vouchers to subsidize rental costs and enhanced access to community based services [86]. A study exploring the longitudinal changes in housing accessibility among clients with various types of disabilities receiving grants for housing adaptations in Sweden found significant improvements in accessibility and usability of housing [76]. Additionally, having financial incentives for different housing provisions and incentives for individuals to stimulate relocation were highlighted in a Swedish study involving housing stakeholders [88]. In particular, subsidies targeting the improvement of current housing stock and interventions focusing on accessibility could be advantageous for housing accessibility [88]. Another study explored housing adaptation grants among a sample of people with various disabilities in Japan noted they were implemented amongst 15.6% of their sample [53]. Meanwhile, Fallon and Price [81] examined the preferences and satisfaction for aging-in-place amongst a sample of people with disabilities in the US who lived in subsidized housing (i.e., low-income housing tax credit program) and explained that residents prioritized affordability and safety. Another financial incentive, the Mountaineer Mortgage Plus Renovation program, offered in a rural area in Ohio US, allowed for the cost of a renovation or home improvement within a mortgage loan, enabling people with disabilities to make necessary modifications to live in their own homes [100].

#### Effective communication and collaborative partnerships

Seven studies explained how having effective communication and/or collaborative partnerships could reduce barriers to acquiring accessible housing [23,54,69,79,88,96,100]. For example, in a UK-based study, Nord et al. [69] explored what constitutes good communication in designing home bathroom adaptations (e.g., new layout and non-slip flooring) and found that a client’s trust in their occupational therapist was important for establishing and maintaining a successful dialogue during the housing adaptation process [69]. Other researchers discussed the usefulness of having knowledge about housing adaptations, including knowing who to contact for facilitating the process, amongst a small sample of adults with functional limitations in Sweden [79]. Participants in this particular study described the benefits of having a collaborative team of professionals (along with input from the client) to develop the best solutions for housing adaptations [79].

Another element of reducing barriers to accessible housing involved effective communication including connecting people with disabilities to accessible housing, often through a matching process. For example, effective communication was also highlighted in a UK-based study with families raising a child with a disability where some reported that being listened to and respected were important components of the housing adaptation process [96]. In another study, applicants for accessible and adapted homes in the UK used a matching approach, which they found helpful, especially when they had a single point of contact to assist them through the process, accessing up-to-date property information and the option for virtual viewings [23]. Effective matching of disabled housing applicants to accessible household properties included renting vacant properties, recovery of properties no longer occupied by a disabled person, nominations through registered social landlords, and renting newly built accessible units [23].

Having collaborative partnerships was another salient aspect of enhancing accessible housing. For example, in interviewing housing stakeholders in Sweden, Heller et al. [88] explained that having effective dialogues and collaboration with various stakeholders could help enhance accessible housing. Another study, focusing on government representatives in Australia, similarly emphasized the importance of developing partnerships for viable accessible housing options and understanding contemporary responses and shared learning amongst key stakeholders [54]. Further, Danko [100] described that developing collaborative partnerships (e.g., builders, money lenders, landlord associations, major building suppliers), consumer guides, model programs (e.g., especially disseminating information about universal design and accessibility), and incentives with housing professionals could help reduce barriers to obtaining accessible housing [100].

### Theme 2: Policies influencing accessible housing

Seven studies in our review focused on policies influencing accessible housing for people with disabilities [54,82,88,98,100,104,105]. For example, Dunn [104] described the evolution of government implemented independent living policies for people with disabilities in Canada and highlighted that barrier-free housing, which is currently in short supply, can assist people with disabilities to live independently. In another study, Semeah et al. [82] found that certain housing policies (e.g., Fair Housing Amendments Act, reasonable modifications, and subsidized housing) facilitated access to rental housing among US veterans with disabilities. Additionally, Danko et al. [100] highlighted that the Community Reinvestment Act in West Virginia, US could facilitate low income individuals with disabilities to obtain accessible housing. Under this Act special programs for people with disabilities are not needed because the majority of banks within this region providing service to low-income individuals, including people with disabilities. Moreover, people with disabilities and their families, living in a rural US state, suggested that enhanced building code enforcement could help to reduce barriers and facilitate access to accessible housing [100]. Another US-based study described how the existence of a policy entrepreneur (i.e., those who work within government to advocate for policy innovations) in a city was positively associated with innovation in housing adoption [98].

In a study exploring how municipalities in Sweden addressed housing accessibility issues and the types of policy solutions considered among housing stakeholders, it was reported that housing accessibility involved renovations and maintenance, individual adaptations, and collaboration with private housing stakeholders regarding housing provision [88]. In a Chinese-based study explored factors that facilitated the adoption of policy innovation on major housing adaptation by provincial governments and found that diffusion mechanisms could either help or hinder the adoption of major housing assessment policy innovations by governments [105]. Moreover, a study [54] examined factors for decision-making and service impacts related to housing and support for people with disabilities in Australia and New Zealand and discovered that insurers influence decisions to fund housing. Specific factors influencing their decisions included understanding demand, working within legislation and rules and individual needs [54].

### Theme 3: Interventions to enhance accessible housing

Twenty-eight studies focused on interventions to enhance accessible housing for people with disabilities, which included home modifications [8,43,55,68,70–74,77,84,85,91,99,101,106–109], smart homes, mobile applications and other experimental devices [4,49–51,65,92,94,110,111].

#### Home modifications

Nineteen studies focused on home modifications to enhance accessibility to enable independent living [8,43,55,68,70–74,77,84,85,91,99,101,106–109,112]. We outline below common household adaptations and factors affecting their implementation.

Twelve studies described common household modifications to enhance accessibility, which often involved entrances, bathrooms, kitchens and bedrooms [8,43,55,68,70–72,77,91,101,106,107]. For example, a study focused on people with mobility disabilities in Thailand, Sukkay [106] found that bathrooms and bedrooms were the two most important rooms to adapt [106]. Meanwhile, Goodwin et al. [8] discovered that common home modifications, among a sample of Australians with mobility impairments, included step-free entrances, wider internal doors and corridors, level access (i.e., no stairs throughout the home) and increased bathroom space. Other accessible household design features included entrance to outdoor spaces, larger room sizes, non-slip flooring, and heights of features and appliances around the home [8]. Similarly, in their study of older adults with spinal cord injuries in Sweden, Norin et al. [68] found that the kitchens (i.e., wheelchair accessible stovetops), main entrances (i.e., ramps) and hygiene areas were common locations for housing adaptations. Further, occupational therapists who conduct home assessments in Australia highlighted that the most important housing features included step-free showers, grab bars, and reinforced bathroom walls, as well as having bedroom space on the ground floor [91]. Similar findings were shown in Bishop et al.’s [72] study where they evaluated the prevalence of housing modifications among American adults with multiple sclerosis and found that the most common modifications involved the bathroom (i.e., grab bars and shower and toilet modifications). Other home modifications noted in this study included entryways (i.e., ramp, grab bars, porch/deck modifications) living area, interior stairs (i.e., lift chairs and elevators), doorways (i.e., widening and lever handles), bedroom (i.e., expanding room or moving room to ground floor), kitchen (i.e., lowering or modifying the cabinets and countertops) and flooring (i.e., replacing carpet with hardwood) [72]. Moreover, people with mobility disabilities in the US reported that changes to the bathroom were the most frequent home modification followed by changes to facilitate cleaning, improve safety, home entrance and mobility within the home [77]. Likewise, for older adults using a walker in New Zealand, Kuboshima and McIntosh [55] highlighted the following design elements and considerations for accessible home modifications: layout of space to avoid needing to turn around in small enclosed spaces, removing ground-level floor differences, minimizing interior doors, avoiding sharp corners, locating door handles, sufficient space in bathrooms and garage, and L- or I-shaped kitchens. Similar renovations were noted among a sample of Koreans with physical disabilities where most home renovations involved safety handles and grab bars, removing door sills and stepped pulleys, replacing wallpaper and finishing materials. Interestingly, this study noted more requests for lighting, thermal environments and improved humidity control in rural areas than urban [43].

Common household modifications differed slightly for people with vision loss. For example, older adults with vision impairments in the UK used adaptive strategies using color contrast on steps and around light switches, and underfloor heating to help reduce the risk of tripping and falling [70]. Participants in this study familiarized themselves with their new surroundings by using techniques such as mind mapping, tactile stickers, removing trip hazards, installing non-slip flooring and eliminating door saddles [70]. The importance of lighting in the home for people with sight loss was examined from practitioners’ perspectives where they emphasized that lighting should be appropriate, sufficient, even, adjustable, sustainable, energy efficient, simple and adaptable [101]. Additionally, for those with visual impairments in the UK, Rooney et al. [107] explored the perceived suitability and effectiveness of lifetime homes (i.e., aging-in-place strategy) and found they offer benefits (e.g., future-proofing features, extra space, and sight loss features). Moreover, Kutintara et al. [71] designed and evaluated a kitchen for people with visual impairments in Bangkok and discovered that people attending cooking courses were able to cook safely in the kitchen. Participants reported they preferred sliding cabinet doors with a shelf inside and D-shaped drawer pulls in addition to contrasting colored strip edges on the kitchen counter [71].

Eight studies described factors affecting household modifications to enhance accessibility [72–74,84,85,93,99,109]. For example, Aplin et al. [85] interviewed participants with various disabilities (and other family members and caregivers) in Australia to understand what aspects of their home environment impact home modification decisions. They reported four key dimensions commonly affecting home modifications, including personal (i.e., safety, privacy, freedom, independence), societal (i.e., costs, service provider and government standards for public access, visitability), physical (i.e., space and dimensions within the home), and temporal (i.e., health status, future growth of family). They also noted that social (i.e., visitability) and occupational (i.e., self-care and domestic activities) dimensions were additional factors impacting home modification decisions [85]. Similarly, a Spanish-based study explained that environmental, economic and functional factors are most relevant in determining adaptations [93]. In particular, their study found that households from bigger cities, richer regions and people with more severe disabilities were more likely to spend money on home adaptations [93]. De-Rosende-Celeiro et al. [74] explored the role of environmental factors in predicting the implementation of bathroom adaptations among older adults with disabilities in Spain and found that social functioning was significantly associated with adaptations. Moreover, people with greater degrees of mobility limitations had an increased likelihood of making a housing modification [72].

Meanwhile, in a small sample of parents of disabled children in Australia, they explained how their decisions to modify their house and purchase equipment were made to enhance their child’s independence and included installing a sensory garden, a spa to enhance physiotherapy treatment, adapted kitchens and bathrooms, and moving to a more accessible home that allowed freedom of movement and access to the community [84]. Another study explored people with physical disabilities in Thailand and noted that people who had a lower extremity impairment or poor balance were more likely to implement housing adaptations [109]. Further, Luther et al.’s [73] study identified five client profiles (based on age and extent of disability) for housing adaptations among a sample of older adults with disabilities in Sweden to understand how home based interventions could be delivered more effectively. In particular, they emphasized the diverse needs of people applying for housing adaptations and the importance of having client profiles to guide professionals on how to differentiate home-based interventions [73].

Another factor affecting home modifications included enhanced training for those who design accessible housing. For example, two studies explored training among architectural students who engaged in a valuable universal design learning exercise with user-experts and explained that it shaped their thinking about universal design and meeting the needs of elderly and disabled clients, as well as their aim to enhance accessible housing [99,113].

#### Smart homes, mobile applications and other experimental devices

Six studies reported on the implementation of smart homes to enhance accessibility [49–51,94,110,111]. For example, Ding et al. [50] explored practitioner’s perspectives on how smart home technology is becoming affordable and relevant for improving environmental control and independence for people with disabilities in the US. They highlighted several common practices relating to smart home service delivery (i.e., importance of client-centered approach, informal service delivery process, trial and error of setting up technology). Their study also emphasized factors affecting smart home service delivery including the influence of client familiarity with technologies and existing use of technology on service delivery, the importance of conducting needs and ability assessments, demonstrating or trialling the technology, client training and device customization [50]. Similarly, Oyegoke et al. [110] developed an innovative smart solution to streamline the housing adaptation process for people with disabilities in the UK. Their “Adapt-ABLE” approach used optimization techniques through an information system allowing for the development of a preventive measure that can access a suitability index of homes for the occupants. In Tayyaba et al.’s [111] study focused on “internet of things” devices for smart homes for people who are blind, they reported that a model smart home with sensors and antennas can generate warning signals about obstacles and help the user to navigate their home safely.

Kosmyna et al. [94] conducted an experiment to assess a smart home control mechanism involving the use of a brain-computer interface among a sample of young adults in France. Their study uncovered that users with disabilities could control lighting, television, coffee machine, and shutters in the smart home (i.e., 81% accuracy versus 77% task accuracy for those without a disability) [94]. Meanwhile, Chen et al. [49] explored the impact of an eyeglass type infrared based home appliance system for people with spinal cord injury and tetraplegia and they found no significant differences in the accuracy and average time cost of the control and experimental groups. They noted that their novel home appliance control system had advantages of convenience, accuracy and sanitation to allow people with spinal cord injury to live more independently [49]. An additional study evaluated the treatment of a smart home enabled by intelligent solutions among a sample of people with various disabilities in Slovenia and found statistically significant improvements in functional independence scores and occupational performance scores [51].

Mobile applications also helped to enhance accessible housing, as outlined in two studies [4,92]. In particular, one study by Helle et al. [92] combined two separate apps that targeted professionals and seniors to improve housing accessibility and housing provisions in a sample of older people in Sweden. They described the development of a user-friendly and acceptable prototype that included design features that differed between professionals (i.e., wanted a design that supported extended functionalities) and seniors (i.e., who wanted a simple and easy to use interface) [92]. In another study, Jonsson et al. [4] co-designed and evaluated a new decision support system to inventory decision-making for improved accessibility for multi-family housing in Sweden and highlighted that their app has potential to support multi-usable professional inventories of environmental barriers, and areas for accessibility improvements.

One study described the development and implementation of an experimental device to enhance accessible housing. Specifically, Mattie et al. [65] explored how a sample of people with mobility disabilities in Canada viewed existing home access solutions that they trialed such as stairs, a ramp, a platform lift, a stair glide lift and a newly designed experimental device (called ARISE). They found that over 80% of participants were satisfied with the ramp and platform lift. They underscored home access features that a majority of participants (90%) considered important: ease of use, ability to use independently, safety and security [65]. Most participants rated the newly designed prototype as their most preferred device [65].

### Theme 4: Impact of accessible independent housing

As a secondary outcome explored in this review, we observed that 16 studies described the impact of accessible housing for people with disabilities. In particular, having household adaptations helped people with various types of disabilities to enhance independence [8,12,23,43,49,50,67,75,79,83,86,87,104], safety [12,50,79,87], quality of life [8,11,23,87,109] participation [12,79,83], social connections [50], community integration [8,74,75], improved health and well-being [8,67,75], decreased physical environmental barriers and decreased dependence on mobility devices [76].

## Discussion

Scholars have considered a range of promising practices, policies and interventions aimed at enhancing accessible independent housing for people with disabilities. Focusing on this topic is salient because access to adequate housing is a fundamental human right [26] and an essential element that can enable independent living and community participation and is closely linked with enhanced health and wellbeing [13,14,16]. Most previous reviews that have considered barriers to obtaining accessible housing have not focused on independent housing; rather they have focused on barriers to other types of housing such as group homes and supportive housing.

Our review highlighted several practices regarding accessible independent housing such as enabling home modifications and removing barriers to obtaining accessible housing at both the societal and individual levels through such things as increasing housing supply, financial incentives, advocacy, effective communication and partnerships. Our findings align with other research showing that home modifications can play a key role in addressing some of the consequences of inaccessible housing [11]. Previous research has consistently shown that increasing awareness about the needs and rights of people with disabilities can help to enhance attitudes and behaviours towards them [114]. Additionally, people with disabilities could also benefit from increased awareness of (and more direct connections to) potential accessible housing options and financial incentives that are available to them [72]. Previous research on accessible housing has highlighted the importance of effective communication between housing stakeholders and people with disabilities, especially involving the latter in the co-design of house modifications [18]. Other research similarly emphasizes the importance of partnerships and collaboration between housing stakeholders and people with disabilities and take their design preferences into consideration [33]. It is essential to engage people with disabilities in program and policy planning to ensure that accessible housing solutions and adaptations meet their needs. It is also evident from the findings of our review that much further advocacy, training and education regarding accessible housing provision and its benefits is critically needed across a range of housing stakeholders (e.g., builders, contractors, planners, designers, architects, real estate professionals, landlords, bank employees, policy decision makers, clinicians doing home assessments). Such training could equip these stakeholders to develop more accessible housing designs, and more streamlined processes for finding and obtaining accessible independent housing and, in turn, they could produce enhanced accessible independent housing options that many people with disabilities desire [100]. Further, it was interesting to note that most of the financial incentives that were described in the studies in our review targeted individuals with disabilities while much less attention was paid to potential incentives for builders and other housing stakeholders to provide accessible housing.

The findings of our review drew attention to some policies influencing the availability of accessible housing, such as fair housing acts, and policies related to modifications and subsidized housing for people with disabilities. Although we noticed many descriptions of policies for accessible housing in the course of our review, most of them were not evaluated. Other research highlights how policies to increase the supply of accessible housing are limited and there is often resistance from developers and builders and other housing stakeholders [41,102]. Additionally, preventive policies such as creating accessibility inventories are essential to ensure that accessibility is addressed before the need arises and strategies targeting relocation are essential [88]. There is also a critical need for key policy decision-makers and other housing stakeholders at various levels of government (municipal, provincial and federal) to work together to develop and implement effective policies that can help to enhance accessible housing options for people with disabilities. Some researchers argue that a wider adoption of universal design approaches to housing is required to enhance the availability of accessible homes [102]. Further work is needed to evaluate how effective existing policies and practices are with respect to their impacts on enabling people with disabilities to obtain accessible independent housing.

Our review has identified some interventions that can enhance accessible housing such as smart homes, mobile applications and experimental devices. Although the interventions highlighted in our review show promise, there is an urgent need for more solutions and interventions to enhance the supply of accessible housing and simplify the laborious processes that people with disabilities must go through to obtain suitable housing (e.g., more matching interventions, the creation of mobile applications that help people with disabilities to find appropriate housing). It will be important to also consider the affordability of accessible housing and whether it is located within safe communities that offer accessible transport options and services [33]. Additionally, a secondary finding of our review emphasized the impact of accessible independent housing on health and wellbeing for people with disabilities, which is consistent with other literature showing how home modifications can help with injury prevention, improved function, independence, physical health and wellbeing and social participation [2,17].

### Limitations, risk of bias and future directions

Although our review was comprehensive and included seven databases without language restrictions, it could be possible that some relevant studies were missed. Also, our review did not include grey literature as it was beyond the scope of our study, and thus we may have missed other potentially relevant accessible housing policies. Second, most studies focused on physical and mobility impairments while there was much less attention to other types of disabilities (e.g., sensory, hearing, vision impairments, brain injury, autism etc.). Future research should explore this gap in further depth. Third, it is important to recognize that perceptions, the treatment of people with disabilities and the availability of supports (including accessible housing and related policies) vary across the 18 countries (mainly higher-income) that were included in our review. It would be worthwhile for further studies to explore the relationships between accessible housing policies and the disability / civil rights laws by country. Fourth, the participant characteristics and methods of the included studies varied widely and the findings of this review should be interpreted with caution. More diverse samples (i.e., gender, racial and ethnic diversity) are needed to explore how socio-demographic characteristics could impact needs and solutions for accessible housing because it is not a one-size-fits all approach. Future research should consider exploring what practices and interventions work best for whom (e.g. considering socio-demographic characteristics), the optimal time to implement them, their cost-effectiveness and in what contexts (e.g., socio-political and individual (rent versus homeowner etc.) they can be applied. Additionally, there was very little research focusing on children and youth with an assumption that people who need accessible housing are elderly. Future research should consider focusing specifically on the housing experiences and perspectives of children and youth because they likely have different needs than older adults. Finally, there was surprisingly little attention paid to affordability of accessible housing. Exploring this aspect further is critical because housing affordability is increasingly an issue of growing concern, particularly for marginalized populations such as people with disabilities [115]. As such, more in-depth research is needed that explores factors affecting home modifications and the ability for people with disabilities to obtain accessible housing.

## Conclusions

This scoping review a range of promising practices, policies and interventions regarding accessible independent housing for people with disabilities over a 20-year period. Our findings uncovered four prominent themes within the literature: (1) removing barriers to obtaining accessible housing; (2) policies influencing accessible housing; (3) interventions to enhance accessible housing (i.e., home modifications, smart homes, mobile applications and other experimental devices) and (4) the impact of accessible independent housing on health and wellbeing. The evidence in this review suggests that there are potential promising practices, policies and interventions to enhance accessible housing through home modifications, smart homes, mobile applications, and experimental devices, which could help to enhance the quality of life of people with disabilities. There is a critical need to continue to advocate and find accessible housing solutions for people with disabilities. Future research should consider focusing on marginalized and minoritized groups who may be more vulnerable to securing accessible housing.

## Supporting information

S1 File Search strategy. (DOCX)

S2 File PRISMA-ScR. (DOCX)
