# Supplementary material for: Accessible independent housing for people with disabilities: A scoping review of promising practices, policies and interventions
Source: PLoS One. 2024 Jan 25;19(1):e0291228. doi: 10.1371/journal.pone.0291228 (PMC10810508; doi:10.1371/journal.pone.0291228)
Supplement: S1 File — (DOCX) [file pone.0291228.s001.docx]

**Supplemental file. Search strategy**

Database: Embase Classic+Embase <1947 to 2022 October 21>, APA PsycInfo <1806 to October Week 3 2022> , Ovid Healthstar <1966 to September 2022>, Ovid MEDLINE(R) ALL <1946 to October 21, 2022>

Search Strategy:

--------------------------------------------------------------------------------

1 disability.mp. (947473)

2 disabled.mp. or exp Disabled Persons/ (326405)

3 disab*.mp. (1362987)

4 functional limitation.mp. (9073)

5 physical impairment.mp. (6997)

6 sensory impairment.mp. (6515)

7 exp Motor Disorders/ or motor impairment.mp. (1321591)

8 hearing impairment.mp. or exp Hearing Loss/ (264851)

9 wheelchair user.mp. (1214)

10 1 or 2 or 3 or 4 or 5 or 6 or 7 or 8 or 9 (2883502)

11 exp Architectural Accessibility/ or accessibility.mp. (425291)

12 universal design.mp. or exp Environment Design/ or exp Universal Design/ or exp "Facility Design and Construction"/ (167175)

13 built environment.mp. or exp Built Environment/ (17770)

14 adaptation.mp. (1013374)

15 environment design.mp. or exp Environment Design/ (36838)

16 smart home technology.mp. (240)

17 11 or 12 or 13 or 14 or 15 or 16 (1524353)

18 housing.mp. or exp Housing/ (188042)

19 house.mp. (248373)

20 home.mp. (1130817)

21 exp Residence Characteristics/ or living environment.mp. (161436)

22 independent living.mp. or exp Independent Living/ (51169)

23 18 or 19 or 20 or 21 or 22 (1638992)

24 10 and 17 and 23 (9426)

25 limit 24 to yr="2007 -Current" (5941)

26 limit 25 to human (5642)

27 remove duplicates from 26 (3649)

***************************
